# Book Reviews

**Published:** 1907-12

**Authors:** 


### BOOK REVIEWS.

———oOo———

### THE USE AND ABUSE OF CARDIAC STIMULANTS.

Hare,—(Therapeutic Gazette.)

In this article the author discusses the common disregard of certain essential details concerning the action of cardiac stimulants. Physicians themselves probably suffer more as a class from this abuse. The "tired heart" commonly existing among physicians usually receives at their hand excessive doses of digitalis instead of the indicated rest. Strong coffee and other adjuncts are also self-prescribed, causing an increase of the cardiac disorder. Another erroneous use of cardiac stimulants is their employment in a state of undue excitation, in which condition cardiac sedatives are needed. Not uncommonly cardiac irregularity calls for small doses of aconite or veratrum ciride. Again a patient with feeble heart receives digitalis when in reality the cause of the feebleness lies in a degenerated heart muscle, which is incapable of gaining any advantage from this drug. In fact by contracting a blood vessel digitalis increases the labor of the heart. Under these conditions strophanthus or cactus, the action of which is cardiac, but slightly if at all vascular, should be used."—Interstate Medical Journal.

This is one of the numerous instances in which cactus is advantageously used. The expressions of the medical profession on Cereus Grandiflorus and Cactina Pillets, which truly presents the therapeutic properties of the drug in the highest form, are very encouraging. It seems that any drug that offers assistance in cardiac complications, and especially if it is devoid of the objectionable features of stronger cardiac remedies, should command the earnest attention of the bedside practioner.

### VISITING & POCKET REFERENCE BOOK -PERPETUAL-

—1908—

J. H. CHAMBERS CO., Publishers,

St. Louis, Mo.

Revised and enlarged, handsomely vellum bound, lapel, pocket size. Price 50c. Condensed,—at the same time sufficiently elaborate to give such information required in a book of this character. Convenient to carry in the pocket; containing 128 printed and blank pages.—SYNOPSIS of CONTENTS: Table of signs, How to keep visiting list,—Obstetrical Memoranda,—Clinical Emergencies,— Artificial Respiration,— Poisons and Antidotes,—Dose Table,—Important Incompatibles; Ruled printed pages for:—Weekly Visiting List,—Memoranda,— Nurses Addresses,—Clinical Record,—Obstetric Record,—Birth Record,—Bills Rendered,— Cash Received,—Miscellaneous Memoranda,—Death Record,— Vaccination Record,—Articles Loaned,—Cash Loaned.

The Publishers will mail copy post-paid on receipt of 24-2c stamps.

THE PRACTITIONERS VISITING LIST for 1908. An invaluable pocket-sized book containing memoranda and data important for every physician, and ruled blanks for recording every detail of practice. The Weekly, Monthly and 30-Patient Perpetual contain 32 pages of data and 160 pages of classified blanks. The 60-Patient Perpetual consists of 256 pages of blanks alone. Each in one wallet-shaped book, bound in flexible leather, with flap and pocket, pencil and rubber, and calendar for two years. Price by mail, post-paid, to any address $1.25. Thumb-letter index, 25 cents extra. Descriptive circular showing the several styles sent on request. Lea Brothers Co., Publishers, Philadelphia and New York.

Being in its twenty-second year of issue, the PRACTITIONERS' VISITING LIST embodies the results of long experience and study devoted to its development and perfection.

It is issued in four styles to meet the requirements of every practitioner: "Weekly," dated for 30 patients; 'Monthly,' undated, for 120 patients per month; "Perpetual," undated, for 30 patients weekly per year, and "60 Patients," undated, for 60 patients weekly per year.

The text portion of the PRACTITIONERS VISITING LIST for 1908 has been thoroughly revised and brought up to date. It contains, among other valuable information, a scheme of dentition; tables of weights and measures and comparative scales; instructions for examining the urine; diagnostic table of eruptive fevers; incompatibles, poisons and antidotes; directions for effecting artificial respiration; extensive table of doses; an alphabetical table of diseases and their remedies, and directions for ligation of arteries. The record portion contains ruled blanks of various kinds, adapted for noting all details of practice and professional business.

